# Annotations

**Published:** 1894-11-03

**Authors:** 


### ANNOTATIONS.

### Does Baking Sterilise a Loaf?

It has been so commonly taken for granted that cooking kills all organisms, and that what is purified by fire is pure, that the observations of Dr. Waldo on the bacteriology of bread are of considerable though somewhat iconoclastic interest. He finds that the process of baking is by no means necessarily destructive of microbic life, and that various organisms retain their vitality in loaves as they leave the baker's oven. Experiments as to the temperature attained in the process of baking showed that the average maximum reached in the middle of an ordinary quartern loaf varied from 163·4 to 186·8 degrees Fahr., and in small loaves from 186·8 to 203 degrees Fahr. This is clearly insufficient to kill many forms of life, and by making bacteriological cultures from the interior of freshly-baked loaves many forms of bacteria were found to have survived. No doubt the greater number of the bacteria present in the dough are destroyed by baking, but the evidence collected by Dr. Waldo shows that this is not always the case, and that there is an evident relation between dirty dough—or, in other words, dirty bakeries—and the number of germs found living in the baked loaf. The character of the loaf seems not unimportant in regard to its freedom from microbic life; the large, somewhat underbaked loaf, dear to Londoners under the name of household bread, being much less completely sterilised than the small, well-baked tinned loaves. In fact, many of the latter were found practically sterile.

### Milk Diet in Bright's Disease.

The interest taken by the public in such details of the Czar's illness as have been published has brought into renewed prominence the use of milk diet in the treatment of chronic Bright's disease. Useful as this method of treatment undoubtedly is, the fact should be remembered that it is by no means the panacea which some people would have us believe. As with every other form of treatment, it must be fitted to the case. First of all we must entirely get rid of the idea that an exclusively milk diet is one which lightens the burden on the kidney by lessening the nitrogenous excretion. As regards solid constituents milk is a very nitrogenous food, and Hale White has shown that, taking actual diets in use at Guy's Hospital, the milk diet, of three pints of milk only, contains more than two-thirds of the proteids contained even in the full diet. The fact is that in regard to the kidneys, and more especially to the heart and blood vessels, it is much more important to consider what happens to the food during its digestion than what it is made of before it is eaten. The subject of diet is inseparably linked with that of digestion. The injurious effect of meat in some cases of Bright's disease depends much more upon the products brewed in the course of its imperfect metabolism than on any strain thrown on the kidney by excess of nitrogenous excretion; and the benefit of milk would seem to arise chiefly from its diuretic action and from its being, as usually taken, a comparatively filling form of low diet. All are agreed as to the usefulness of milk in cases of acute disease attended with dropsy and scanty urine. It is otherwise, however, in the later stages of chronic Bright's disease, in which the tendency to death is really not a matter of kidney but of heart. In these cases very often there is a free diuresis to begin with, and if any deficiency in that direction occurs it is more from failing blood supply than from any exacerbation of the malady. True it is that milk, which as regards bodily activity is a low diet, does very well as part of that "life on a lower plane" which is necessary for patients suffering from this disease, but when people become really ill, and when even on that lower plane the heart fails to overcome the increasing difficulties of the circulation, a more generous diet is often necessary. Dr. Hale White's conclusion is definite that on the whole an ordinary full diet is best for this disease, and that patients feel better and stronger upon it. Dr. Ralfe says that milk diet is best in cases of acute or subacute nephritis, but that where degenerative changes have taken place it is better to give a more solid and stimulating food. The outcome then, speaking generally, is this: that in acute cases milk diet should be given; that in chronic cases milk diet is useful if well digested and combined with a simple careful life, that it is often more easily digested, and no harm is thereby done to the kidney, when combined with farinaceous food; but that when anæmia threatens and the heart tends to fail the centre of interest is shifted from the kidney to the circulatory system and a more supporting regimen must be adopted. As a final warning we would add that in any case where milk alone is ordered care should be taken to ascertain that it is properly digested, for if not milk diet becomes a very low diet indeed, consisting chiefly of water, fat, and sugar.

### Doctors and Relieving Officers.

A subject of some importance has recently come before the Board of Guardians at Cork. A complaint was lodged against one of the relieving officers for having refused to send a patient to hospital which one of the medical officers had certified as fit for hospital treatment. Considerable surprise and some indignation has been expressed at what is spoken of as the high-handed proceedings of the relieving officer in, as it is said, treating the certificates of the dispensary medical officers "with contempt." But if we calmly look at the facts, and consider what are the duties of the respective officials, it is clear that there is but small excuse for the excitement. The error arises from looking upon the doctor's certificate as an order for admission. It is no such thing, and no slur is necessarily cast upon the doctor by not acting upon it. The poor law would be unworkable if the outside medical officers were able by means of the certificate to exercise concurrent jurisdiction with the relieving officers in the application of "the test" of ordering removal to the workhouse, especially as in many cases the medical officer would to a certain extent be an interested party in such removal. If the dispensary doctor could fill the house with "chronics" a nice time the infirmary authorities would have! The fact is the discretion as to removal rests with the relieving officer, and the doctor's certificate is merely to tell him whether the case is fit for removal or not.

